# Development of the Applied Mindfulness Process Scale as a Process Evaluation Measure for Mindfulness Practice in a Chinese Context

**DOI:** 10.3389/fpsyg.2022.848787

**Published:** 2022-03-11

**Authors:** Yitong Jia, Yitian Yan, Wen-Xin Shi, Ge Meng, Xinqi Zhuang, Yin-Ping Zhang

**Affiliations:** ^1^School of Nursing, Xi’an Jiaotong University Health Science Center, Xi’an, China; ^2^Zonglian College, Xi’an Jiaotong University Health Science Center, Xi’an, China

**Keywords:** mindfulness, applied mindfulness process scale, reliability, validity, scale

## Abstract

With the rising popularity of mindfulness practice, it is necessary and crucial to evaluate mindfulness using comprehensive and objective measures. The instruments to assess mindfulness in China mainly evaluate mindfulness as a state or trait mode. Few process measures have been developed to clarify effective therapy benefits of the alterations obtained using mindfulness practice. Therefore, this study aims to adapt the Applied Mindfulness Process Scale (AMPS) into Mandarin and explore in detail the reliability and validity of this novel-translated measure. Following cross-cultural modification for original AMPS into Mandarin as per established guidelines, psychometric evaluation was performed on a cohort of 234 Chinese adults. Construct validity was analyzed through exploratory factor analysis (*n* = 115), together with confirmatory factor analysis (*n* = 119). Reliability was assessed by internal consistency together with test-retest reliability. Findings indicated that the internal consistency was high, with Cronbach’s alpha being 0.936. The principal component analysis led to a three-factor structure that explained 67.374% of all variations. The three-factor model was consistent with the original scale model. Based upon confirmatory factor analyses, all fitting indices satisfied the standard, which showed a close fit to the data. Therefore, the newly multi-culturally modified AMPS has sufficient validity, test-retest reliability, together with internal consistency. Chinese AMPS may offer researchers and clinicians a psychometrically optimized tool for evaluating the application of mindfulness and change process within mindfulness-based interventions (MBIs) in Mainland China.

## Introduction

Mindfulness was typically described as the awareness that emerges by intentionally bringing one’s attention, in a non-judgmental manner, to the internal and external experiences that exist in the present moment (Kabat-Zinn, 2003). With the advances in standardized mindfulness-based interventions (MBIs) and its proper implementation, mindfulness has been recognized and spread rapidly in Western psychology (Gu et al., 2015). MBIs consist of a mixture of mind-body practices applied for enhancing mindfulness consciousness, represented through the momentary-based non-judging attention being freed from the abstraction and focus of cognitive emotions (Kabat-Zinn, 2003). Considerable research has shown that MBIs are highly therapeutic for multiple conditions/wellbeing states among diverse populations (Hofmann et al., 2010; Eberth and Sedlmeier, 2012; Khoury et al., 2013), including anxiety (Hofmann et al., 2010; Green and Bieling, 2012; Hinton et al., 2013), depressive relapsing risks (Teasdale et al., 2000; Kuyken et al., 2009), depression (Strauss et al., 2014), stress (Chiesa and Serretti, 2009), chronic pain (Grossman et al., 2007), quality of life (Kuyken et al., 2009; Godfrin and Van Heeringen, 2010), together with psychological/emotional distress (Ledesma and Kumano, 2009; Xu et al., 2016). Furthermore, MBIs have demonstrated to be effective through specific psychopathological alterations, including cognitive biases, affective dysregulation, and interpersonal effectiveness (Brown et al., 2007; Bullis et al., 2014; Curtiss et al., 2017).

Due to the rising popularity of MBIs, proper assessment of mindfulness is essential. The development of reliable, validated instruments gives trainers the possibility to evaluate their interventions, together with researchers being able to analyze potential psychological processing mechanisms using mindfulness. Some self-report state and trait measures are already established, including the Mindful Attention Awareness Scale (MAAS) (Brown and Ryan, 2003), Freiburg Mindfulness Inventory (FMI) (Walach et al., 2006), Kentucky Inventory of Mindfulness Skills (KIMS) (Baer et al., 2004), Five-Facet Mindfulness Questionnaire (FFMQ) (Baer et al., 2006), Cognitive and Affective Mindfulness Scale (CAMS) (Feldman et al., 2007), together with the Toronto Mindfulness Scale (TMS) (Lau et al., 2006). Notwithstanding the proliferation of instruments for mindfulness evaluation, all the tools mentioned above cater for mindfulness assessment through an outcome perspective, not from a processing perspective. Such a methodology kept limited understanding of how mindfulness-based skills and practices were applied in daily life when encountering life stressors. For the purpose of elucidating treatment mechanisms for alterations obtained using mindfulness practice, it was essential to develop a process measure. Measuring the process of mindfulness practice would enable researchers to recognize more effectively the specific mechanistic pathways by which mindfulness and corollary advantages are attained within those involved in MBIs, together with its implementations in day-to-day living situations (Chiesa et al., 2014). The existing scales used to measure the process of mindfulness practice mainly consist of the 7-item Mindfulness Process Questionnaire (MPQ) developed in 2012 and the Applied Mindfulness Process Scale (AMPS), developed in 2016. The MPQ identifies situations in which an individual shifts to a higher mindful state and guides the participant to note how they focus on the process of becoming mindful, instead of the “success” in achieving mindfulness (Erisman and Roemer, 2012). However, the MPQ did not emphasize the application of mindfulness practice. It was difficult for researchers to use this instrument to explore the mechanism of mindfulness practice, which could limit their design of MBI programs. The AMPS is a 15-item process measure developed by Michael J. Li and colleagues (Li et al., 2016). Compared with the MPQ, the AMPS contextualized the application of mindfulness. Every individual AMPS entry was designed to reflect the application of mindfulness in coping with negative states, challenging times, and daily stresses through positive/negative emotional regulation and decentering. The newly well-designed AMPS can be applied to evaluate not only the application of mindfulness but also the change process within mindfulness practice.

In recent years, research on mindfulness practice or MBIs has shown an increasing trend in China. Although several self-report state and trait measures, such as MAAS, FFMQ and FMI, had been validated in Chinese culture (Chen et al., 2012; Chen and Zhou, 2013; Meng et al., 2019), few process measures on the application of mindfulness practice have been developed in the Chinese context. Consequently, the development and psychometric adaptation of a Chinese AMPS can offer a novel angle for identifying a spectrum of therapy-aimed facets of MBIs for Chinese researchers and further lay the foundation for research on applying MBIs in China. In this study, we cross-culturally adapted the AMPS as a psychometric process measure in China to capture the implementation of practical, daily-life mindfulness practice and consequently evaluated its psychometric properties.

## Materials and Methods

### Procedure and Participants

Psychometric properties for the translated scale were evaluated using a cross-sectional survey. Two hundred thirty-four residents were recruited using convenience sampling from multiple (5) districts of Xi’an, the capital of Shaanxi Province in Northwest China. The city was selected because it is the largest designated central city in Northwest China and is accessible to the investigators. Participant inclusion criteria included: (a) reaching the age of 18; (b) possessing household registration in Xi’an or the permanent residence is Xi’an; (c) possessing the ability of reading and communication; (d) willing to participate in the study. All participants had signed informed consent following a detailed description of the investigation goals and methodologies involved. Meanwhile, all members of the study cohort were advised that withdrawal from the investigation could be performed at any point during the trial, without any repercussions. Anonymity and confidentiality were assured. Written informed consent was taken from all participants before the study. All procedures performed in studies involving human participants were in accordance with the Ethics Committees of Xi’an Jiaotong University and with the 1964 Helsinki declaration and its later amendments or comparable ethical standards.

### Instruments

#### Applied Mindfulness Process Scale

The original Applied Mindfulness Process Scale (AMPS) was developed in 2016 and comprised three subscales: positive emotion regulation (5 items), negative emotion regulation (5 items), and decentering (5 items). Positive Emotion Regulation consisted of items reflecting mindfulness-based coping mechanisms through re-focusing onto positive emotional experiences and positive re-appraisal of challenging life situations. Negative Emotion Regulation included items reflecting mindfulness-based coping mechanisms through reducing negative emotion. Decentering consisted of items reflecting mindfulness-based coping mechanisms by the separation from negative feelings/thoughts through the consideration of individual mental experience as lacking absolute authenticity (Li et al., 2016). Each item was scored from 0 to 4 (0 = never; 1 = rarely; 2 = sometimes; 3 = often; 4 = almost always). According to psychometric properties testing, the overall AMPS had good internal consistency with Cronbach’s alpha coefficient of 0.91. Comparative fit index (CFI) for the three-factor model was 0.97, Tucker-Lewis index was 0.94, and the root-mean-square-error-approximation (RMSEA) was 0.06, indicating it had a valid psychometric parameter (Li et al., 2016).

#### General Information Questionnaire

We generated the General Information Questionnaire to investigate sociological, demographics-based and health-related status. The questionnaire included age, gender, educational background, health condition, etc.

### Translation Process

After receiving permission to use this instrument from the copyright holders, the Chinese translation process for AMPS was completed in two steps using a clear guideline (Beaton et al., 2000). Firstly, in line with Brislin’s translation procedure (Brislin, 1970), a committee of two collaborators who were fluent in Chinese/English and proficient in medical psychology separately accomplished their own version of the first AMPS draft. Consequently, a third individual was required to combine both draft versions while also referring to the original scale. This third, combined draft was consequently and blindly back-translated separately by two translators into English, as accuracy assurance.

### Experts’ Reviewing Process

A total of seven experts were recruited for the reviewers’ board, consisting of psychologists, mental health workers, nursing professors, and managers, all with a Master’s degree or higher and with expertise in clinical psychology and scale development. This board assessed the source scale together with all subsequent translated AMPS drafts, verifying the equivalence of phrases within both the initial AMPS and the finalized back-translated AMPS draft. The review board members continued discussing all AMPS items at each stage of reviewing until a consensus was reached. Joint rating for the finalized AMPS was then performed in order to assess the content validity index (CVI), for individual scale items (I-CVIs), together with full scale (S-CVI).

### Pilot-Study

A total of 20 residents recruited through convenience sampling in Northwest China were informed to fill in the Chinese AMPS questionnaire together with delineating any unclear/incorrect items and propose alternatives. The completion timeframe was approximately 5–10 min. An interview was performed to evaluate participant perspectives for each entry and register their suggestions for optimizing the Chinese AMPS. Following the discussion between researchers and participants, all ambiguous issues were fixed. One typical issue concerned the improvement of response rate of the questionnaires and participant fear reduction by changing the order of item 1 (Observe thoughts in a non-attached manner.) and item 2 (Relax my body when I was tense.). Moreover, the translations of some phrases were changed to adapt to the Chinese context. Following this process, the final Chinese version was developed. The 20 participants were asked to complete the scale again after 2 weeks, for assessing test-retest reliability.

### Data Analysis

Statistical Package for the Social Sciences (SPSS) version 20.0 was employed. Statistical description for demographic variables was performed using means, standard deviations (SD), and frequency tables. Content validity index (CVI) was conducted for score quantification on each entry and the total AMPS. The CVI was generated through expert-opinion on relevance ratings for each entry, through a 4-point scale, from 1 (highly invalid) to 4 (highly valid). Any item being designated a 3 or 4 on this 4-point scale indicated that experts have reached a consensus on the relevance of this item. Individual item CVI was consequently generated, with a score of above 0.8 denoted validity (Polit et al., 2007). Internal consistency was generated *via* Cronbach alpha coefficient, whereby values ≥0.7 were deemed to be sufficient (Osburn, 2000). Split-half coefficient reliability was generated through the employment of 50% of all odd/even items. Test-retest reliability was generated through intra-class correlation coefficient (ICC) for AMPS items (Giavarina, 2015), whereby values of 0.60–0.80 were accepted as reliable, with values above 0.80 having excelled in reliability (Chavance, 2008). Individual item validity was identified using item analysis. Unfavorable floor/ceiling influences were deemed to exist if more than 15% of survey-participants achieved the highest/lowest score. Exploratory factor analysis employing principal component analysis (PCA) with oblique rotation was performed for assessing AMPS factor structure (Pryse et al., 2015). Scree plot, Kaiser Criterion (eigenvalue), combined with clinical interpretation, were all used to recognize factor solution. Items were considered relevant if factor-loading coefficients were above 0.40/gathered factors obtained an eigenvalue ≥1.0 (Sapountzi-Krepia et al., 2016). Confirmatory factor analysis (CFA) was also done for verifying factor structure. Expected values of proposed indices were (Batista-Foguet et al., 2004): (a) Chi-squared divided by the degrees of freedom ≤ 3; (b) the root mean squared error of approximation (RMSEA) < 0.08; (c) the comparative fit index (CFI) > 0.90 and goodness-of-fit index (GFI) > 0.90.

## Results

### Sample Characteristics

Table 1 summarizes the participant characteristics. In summary, 234 residents participated in our study, including 128 (54.7%) females and 106 (45.3%) males. The mean age was 38.56 years (*SD* = 10.575). Participants were well-educated, with more than 44% having a bachelor’s degree or higher. The main occupations of the participants included: Peasantry (10.7%), Worker (8.5%), Staff (32.5%), Cadre (8.5%), Retired or unemployed (30.8%), and other (9.0%). There were 141 (60.2%) participants whose monthly household income was more than 3,000 yuan, though 59.0% of the participants were in a state of illness (indicating 59.0% of the participants had acute or chronic diseases) at the time of the investigation.

**TABLE 1:** Demographic characteristics of sample (*n* = 234).

Characteristics	Respondents	n	%
Age (years)	18–40	149	63.7
Mean: 38.56	41–60	85	36.3
SD: 10.575			
Gender	Male	106	45.3
	Female	128	54.7
Ethnicity	Han	229	97.9
	Minorities	5	2.1
Education	Elementary school or lower	40	17.1
	Junior middle school	39	16.7
	High school/Vocational School	52	22.2
	College or higher	103	44.0
Employment	Peasantry	25	10.7
	Worker	20	8.5
	Staff	76	32.5
	Cadre	20	8.5
	Retired or unemployed	72	30.8
	Other	21	9.0
Marital status	Married	132	56.4
	Unmarried	99	42.3
	Other	3	1.3
Monthly household income per person (yuan)	<1,000	37	15.8
	1,000–2,999	56	24.0
	3,000–4,999	53	22.6
	≥5,000	88	37.6
Illness	No	96	41.0
	Yes	138	59.0

### Item Analysis

First, we sorted AMPS items within high-/low-scoring subgroups, whereby the highest-scoring 27% formed the high subgroup, and the least-scoring 27% formed the low subgroup. Consequently, a comparative analysis of the mean individual-item score for both subgroups was performed. Item analysis (Table 2) confirmed variation between the two subgroups with statistical significance (*p* < 0.001). Item analysis was additionally conducted on the subscale. For the high subgroup, the mean scores for decentering, positive emotion regulation, and negative emotion regulation were 21.850 (*SD* = 1.039), 22.383 (*SD* = 1.354), and 21.550 (*SD* = 1.567), respectively. For the low subgroup, the mean scores for decentering, positive/negative emotion regulation were 12.167 (*SD* = 2.203), 13.650 (*SD* = 2.364), and 12.300 (*SD* = 2.036), respectively. These results demonstrated that each item had solid discrimination properties exempted from floor/ceiling effects and, consequently, no entry was excluded.

**TABLE 2:** Item analysis of the Chinese version of AMPS (*n* = 234).

Subscale	Low score group (*N* = 59)	High score group (*N* = 69)	t	*p*
Decentering	12.167 ± 2.203	21.850 ± 1.039	–30.797	<0.001
Positive Emotion Regulation	13.650 ± 2.364	22.383 ± 1.354	–24.833	<0.001
Negative Emotion Regulation	12.300 ± 2.036	21.550 ± 1.567	–27.888	<0.001
Total	38.683 ± 6.072	64.700 ± 3.475	–28.806	<0.001

### Reliability

The summaries for internal consistency, together with split-half reliability for AMPS are illustrated in Table 3. The Cronbach’s alpha on the total questionnaire was 0.936, with the three subscales having a Cronbach’s alpha of 0.887 (decentering), 0.860 (positive emotion regulation), and 0.922 (negative emotion regulation). Split-half reliability for all items of the scale was 0.902, with subscale values ranging between 0.823 and 0.931. Test-retest reliability through ICC was 0.861 for the total scale and 0.841 to 0.860 for the three subscales, which showed a good stability.

**TABLE 3:** Reliability of the Chinese version of AMPS (*n* = 234).

Subscale	No. of items	Cronbach’s α	Split-half	Test-retest
Decentering	5	0.887	0.888	0.841
Positive Emotion Regulation	5	0.860	0.823	0.855
Negative Emotion Regulation	5	0.922	0.931	0.860
Total	15	0.936	0.902	0.861

### Content Validity

All experts agreed that the AMPS was mainly developed to evaluate how participants apply mindfulness practices for coping with stressors and hassles in everyday life. All items had been reviewed by the reviewer board to be “very relevant”/“quite relevant.” Item-level CVI ranged from 0.88 to 1.0. Scale-level CVI reached 0.984, indicating excellent content validity.

### Construct Validity

#### Exploratory Factor Analysis

**FIGURE 1:**
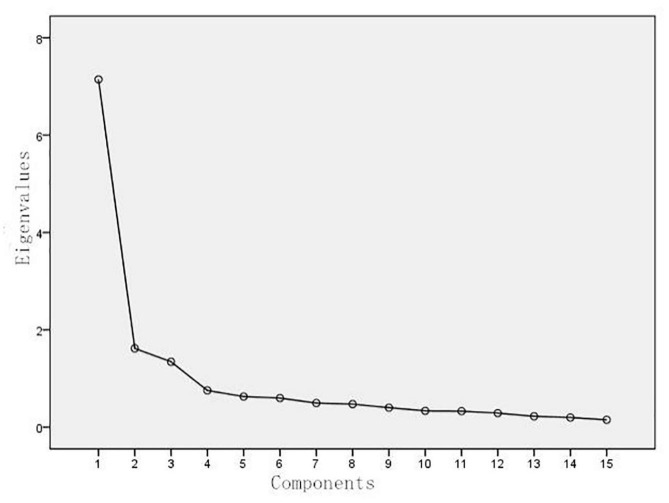
Scree plot of the Chinese version of AMPS.

In this study, the data was divided into two parts randomly. The first 115 samples (SPSS marked as “0” subgroup automatically) were used for exploratory factor analysis. Principle component analysis (PCA) with oblique rotation was employed to assess the factorial structure of the 15-item AMPS. The Kaiser-Meyer-Olkin (KMO) recording of sampling adequacy was 0.881, and Bartlett’s test of sphericity was statistically significant (*p* < 0.01), indicating that such data were appropriate for factor analyses. Following Kaiser’s criterion of extracting factors having eigenvalues >1 (Factor 1 = 7.144, Factor 2 = 1.619, Factor 3 = 1.344) and the scree plot (Figure 1), a three-factor structure that explained 67.374% of the total data variance was revealed through pattern matrix. The communalities of all items ranged from 0.475 to 0.761, which were more than 0.4. Therefore, all items were reserved. Exploratory factor analysis for all items generated factor loading that ranged between 0.615 and 0.823. Item-15 (“see alternate views of a situation”) can correspond to either factor 2 (factor loading: 0.515) or factor 3 (factor loading: 0.665). According to the professional judgment of its content and factor loading, it was finally included in factor 3, the Decentering subscale. The final exploratory factor analysis (EFA) results are illustrated in Table 4.

**TABLE 4:** Factor loadings on items of the Chinese version of AMPS (*n* = 115).

Item No.	Dimension	Item	Factor 1	Factor 2	Factor 3	Communalities
2	D	Observe my thoughts in a non-attached manner			0.727	0.678
3	D	See that my thoughts were not necessarily true			0.823	0.742
12	D	Let go of unpleasant thoughts and feelings			0.693	0.675
13	D	Realize that my thoughts were not facts			0.758	0.696
15	D	See alternate views of a situation		0.515	0.665	0.719
4	P	Enjoy the little things in life more fully		0.615		0.475
7	P	See the positive side of difficult circumstances		0.759		0.639
9	P	Realize that I can grow stronger from difficult circumstances		0.733		0.597
11	P	Be aware of and appreciating pleasant events		0.728		0.580
14	P	Notice pleasant things in the face of difficult circumstances		0.752		0.703
1	N	Relax my body when I was tense	0.791			0.747
5	N	Calm my emotions when I was upset	0.805			0.761
6	N	Stop reacting to my negative impulses	0.793			0.726
8	N	Reduce tension when I was stressed	0.703			0.688
10	N	Stop my unhelpful reactions to situations	0.745			0.680
Eigenvalues			7.144	1.619	1.344	
Variance (%)			47.624	10.790	8.960	
Cumulative (%)			47.624	58.414	67.374	

*D represents Decentering subscale, P represents Positive Emotion Regulation subscale, N represents Negative Emotion Regulation subscale; Responses are on a 5-point Likert scale from 1 never to 5 almost always.*

#### Confirmatory Factor Analysis

**FIGURE 2:**
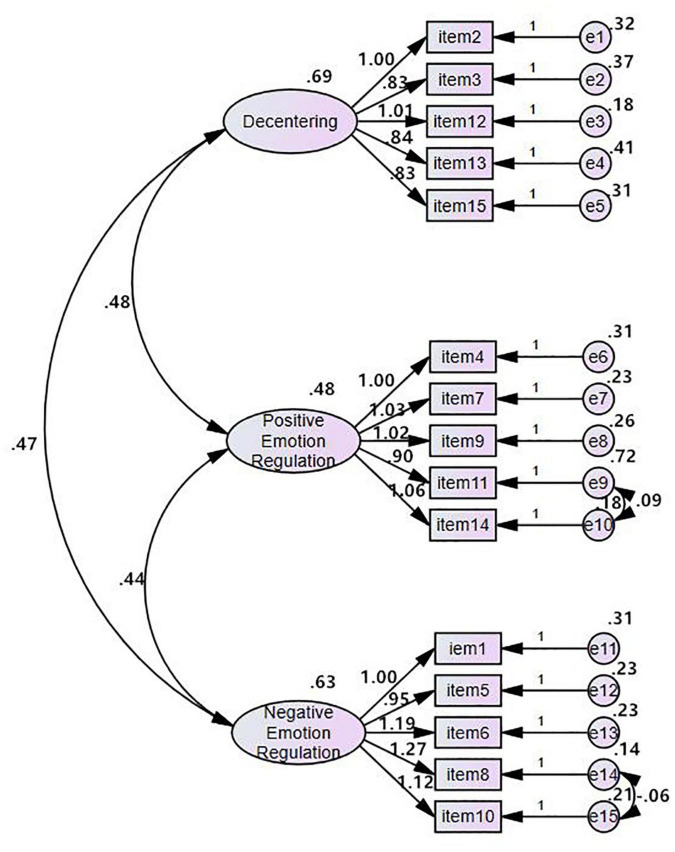
A schematic diagram of standardized model fitting of the Chinese version of AMPS.

A total of 119 samples (SPSS marked as “1” subgroup automatically) were used for confirmatory factor analyses. A three-factor model was established according to exploratory factor analytical outcomes (see Figure 2 and Table 5). Prior to modification, individual item factor loading ranged between 0.83 and 1.27, all above 0.7. All fit indices, except the *p*-value and GFI within the initial model, complied with suggested parameters for satisfactory model fitting. The fit indexes were excellent in the modified model: the *p*-value was 0.093, exceeding 0.05. The indices for GFI (0.902 for the modified model) and CFI (0.987 for the modified model) exceeded 0.90. The RMSEA was 0.042, less than 0.08. Consequently, the correlated three-factor structure contributed an ideal model fit as previously predicted, and its application seems appropriate for the population survey.

**TABLE 5:** The fitting indexes of confirmatory factor analysis of the Chinese version of AMPS (*n* = 119).

Index	Benchmark	Initial model	Modified model
χ^2^/df	<3	1.297	1.208
*p*	>0.05	0.033	0.093
GFI	>0.90	0.890	0.902
CFI	>0.90	0.981	0.987
RMSEA	<0.08	0.050	0.042
NFI	>0.90	0.924	0.931
IFI	>0.90	0.982	0.987

## Discussion

In the present study, we cross-cultural adapted the AMPS into standard Mandarin and explored the reliability/validity for such a novel-translated instrument in China. It was meaningful for Chinese researchers to use the Chinese version of AMPS for analyzing how people employ mindfulness for coping with stress throughout life. All preliminary efforts we conducted ensured AMPS to be designed in compliance with the rules of Standard Mandarin. For this purpose, the forward-backward translating methodology was implemented. In addition, the draft was given to 20 participants for assessing the complexity level in reading and interpretations. According to the pilot results, we had a heated discussion on the translation of some English words and finally reached a consensus. Following these changes, the participants were re-queried, and they claimed they would answer the same way for both items, but the latter construction made more sense in Chinese. Such provisions lead to a semantically, idiomatically, experientially, and conceptually equivalent scale to the original.

Our project also played a crucial role regarding reliability/validity for cross-cultural AMPS. Reliability represents the stability, accuracy and consistency of a measurement (Zhen-qiu, 2011). The Cronbach’s alpha was 0.936 in our 234 residents sample in the northwest of China, indicating a good internal consistency. This result was higher than the result shown in the original version (Li et al., 2016). One reason may be the cultural sensitivity of translation. Mindfulness originated from Southeast Asia and influenced by the regional culture, where the scale is suitable for Chinese citizens. Alternately, such enhanced reliability could be stemming from an increased sample size in this study (234 within our investigation vs. 134 within the original research). Test-retest intra-class correlation was 0.861 for the overall scale, demonstrating that the AMPS is stable over time (across 2 weeks in this study). This adds further information on the reliability of this scale, since test-retest reliability was not measured within the original AMPS validation trial.

Evaluating the validity of the scale can verify the level of compliance between the measured and expected outcomes (Zhen-qiu, 2011). Both content and construct validity were measured for this Chinese AMPS. CVI was employed for content validity analysis. All experts were asked to make comments or suggestions on any items in the inquiry letter of the scale, whereby we would modify any concern only if at least two experts voiced such a concern. The results revealed the majority of items attained 3–4 points. The scale-level CVI reached 0.984, and the CVI for each item was reportedly higher than 0.78, suggesting a satisfactory degree of content validity for the measurement. Both factor analysis and structural equation modeling confirmed the construct validity in the study. Exploratory factor analysis suggested that the extracted three principal components were theoretically equivalent to the initial AMPS structure, accounting for 67.374% of all variance, providing appropriate indices for analyzing the validity of this scale. There were good corresponding relations between Items 1–14 and the expected dimension. Item-15 (“see alternate views of a situation”) can correspond to either factor-2 (Positive Emotion Regulation) or factor-3 (Decentering). This is probably since Chinese people often use the strategy of “look at problems from different angles” to deal with disadvantageous things. By finding the positive aspects in the adverse environment, they change their self-awareness and enhance their positive emotions. However, mindfulness is about attention. We are all mindful to some extent, in each moment. It is an innate capacity. Mindfulness emphasizes simple and efficient routes for cultivating and refining such a capacity and apply it to all aspects of life, not only under adverse circumstances (Kabat-Zinn, 2003). Therefore, According to the professional judgment of its content and factor loading, it was finally included in factor 3.

As an additional study, we conducted CFA to investigate the fit of all three subscales with the overall AMPS structure. The goodness of fit indices in the CFA model was almost achieved, and all fit indices satisfied the standard. Data outcomes of the exploratory/confirmatory factor analyses were consistent; demonstrating the data in our study were consistent with the intrinsic hypothesis. Considering such obtained results, the AMPS consisted of three factors: decentering, positive emotion regulation and negative emotion regulation. It was different from MAAS. The MAAS contained a single factor described as awareness of and attention to the present moment. However, Brown and Ryan found MAAS scores were negatively correlated with negative emotion and positively correlated with psychological wellbeing (Brown and Ryan, 2003), which to some extent supported our findings. Additionally, the decentering dimension was similar to the facets such as “non-judging of inner experience” or “non-reactivity to inner experience” which were presented in other measures (R. A. Baer et al., 2006). This is partly because decentering is identified as an essential component of mindfulness. Our three-factor model is consistent with the original scale model, indicating the Chinese AMPS to have appropriate construct validity. Studies have shown that mindfulness may enable a practitioner to recognize that his/her thoughts might not always be true and that the self might not be consistent with mental experience. Consequently, he/she could apply increasingly accurate situational appraisals, reducing bias caused by cognitive distortions (Mathews and MacLeod, 2005). Simultaneously, modern psychological theories hold that mindfulness can promote positive emotional status. Similarly, Buddhist academics endorsed using mindfulness for promoting a “cultivation of happiness, the genuine inner transformation by deliberately selecting and focusing on positive mental states” (Garland et al., 2010). Furthermore, when facing negative emotions sparked by stressful events, practitioners may use mindfulness to calm themselves down and thereby rein the impulse to react negatively when under stress (Jain et al., 2007). These results also provide a theoretical basis for the construction of the three-factor model of AMPS.

We present the first Chinese version of the AMPS to be developed and psychometrically evaluated. The newly developed Chinese version of AMPS is concise and is a user-centered tool for self-reporting. This scale was designated as easy for users to complete and required minimal/no explanation from the investigator. Completion of the questionnaire took less than 10 min (mean timeframe). Compared with other instruments for measuring mindfulness, AMPS is a process analysis tool that can be adept for use in MBI trials, and can be employed to ascertain how mindfulness will be applied to various therapeutic processes and predict a range of clinical outcomes.

### Limitations

Although the result of the cross-cultural adaption of the AMPS is satisfied, a few limitations were present. Firstly, participants were gathered through convenience sampling in Northwest China, which may have impacted the generalizability of the findings to some degree. However, the sample in our study had a broad range of education levels, employment status, and age range, suggesting that AMPS is understandable and acceptable by the general Chinese population. Secondly, the study did not measure the criterion validity. A psychometric evaluation of AMPS concerning criterion validity should be taken into consideration within future validation studies.

## Conclusion

The Chinese version of AMPS is expressly developed as a process measure to assess how individuals employ mindfulness for MBIs or within daily life. It has important value and can be easily implemented. Our results indicated that the Chinese version of AMPS is a reliable and valid instrument and may thus be used as a process measure to evaluate the application of mindfulness practice in the Chinese context.

## Data Availability Statement

The raw data supporting the conclusions of this article will be made available by the authors, without undue reservation.

## Ethics Statement

The studies involving human participants were reviewed and approved by the Ethics Committees of Xi’an Jiaotong University. The patients/participants provided their written informed consent to participate in this study.

## Author Contributions

Y-PZ and YJ performed the study design and manuscript preparation. YJ and YY performed the data collection and analysis. W-XS, GM, and XZ drafted and edited the manuscript. All authors read and approved the final manuscript.

## Conflict of Interest

The authors declare that the research was conducted in the absence of any commercial or financial relationships that could be construed as a potential conflict of interest.

## Publisher’s Note

All claims expressed in this article are solely those of the authors and do not necessarily represent those of their affiliated organizations, or those of the publisher, the editors and the reviewers. Any product that may be evaluated in this article, or claim that may be made by its manufacturer, is not guaranteed or endorsed by the publisher.
