# Supplementary material for: Development of the Applied Mindfulness Process Scale as a Process Evaluation Measure for Mindfulness Practice in a Chinese Context
Source: Front Psychol. 2022 Mar 11;13:848787. doi: 10.3389/fpsyg.2022.848787 (PMC8963177; doi:10.3389/fpsyg.2022.848787)
Supplement: Supplementary file 1 [file Table_1.DOCX]

**Supplementary Table 1.**The Applied Mindfulness Process Scale (AMPS)

| **I used mindfulness to...** | | **Never** | **Rarely** | **Some**  **times** | **Often** | **Almost Always** |
| --- | --- | --- | --- | --- | --- | --- |
| 1. Observe my thoughts in a non-attached manner |  | 0 | 1 | 2 | 3 | 4 |
| 1. Relax my body when I was tense |  | 0 | 1 | 2 | 3 | 4 |
| 1. See that my thoughts were not necessarily true |  | 0 | 1 | 2 | 3 | 4 |
| 1. Enjoy the little things in life more fully |  | 0 | 1 | 2 | 3 | 4 |
| 1. Calm my emotions when I was upset |  | 0 | 1 | 2 | 3 | 4 |
| 1. Stop reacting to my negative impulses |  | 0 | 1 | 2 | 3 | 4 |
| 1. See the positive side of difficult circumstances |  | 0 | 1 | 2 | 3 | 4 |
| 1. Reduce tension when I was stressed |  | 0 | 1 | 2 | 3 | 4 |
| 1. Realize that I can grow stronger from difficult circumstances |  | 0 | 1 | 2 | 3 | 4 |
| 1. Stop my unhelpful reactions to situations |  | 0 | 1 | 2 | 3 | 4 |
| 1. Be aware of and appreciating pleasant events |  | 0 | 1 | 2 | 3 | 4 |
| 1. Let go of unpleasant thoughts and feelings |  | 0 | 1 | 2 | 3 | 4 |
| 1. Realize that my thoughts were not facts |  | 0 | 1 | 2 | 3 | 4 |
| 1. Notice pleasant things in the face of difficult circumstances |  | 0 | 1 | 2 | 3 | 4 |
| 1. See alternate views of a situation |  | 0 | 1 | 2 | 3 | 4 |

**Supplementary Table 2.** The Chinese Version of the Applied Mindfulness Process Scale

正念实践评价量表

| 在日常生活中，我可以…… | 从未 | 很少 | 偶尔 | 经常 | 总是 |
| --- | --- | --- | --- | --- | --- |
| 当我紧张时，放松我的身体 | 0 | 1 | 2 | 3 | 4 |
| 以旁观者的角度超然地观察自己的想法 | 0 | 1 | 2 | 3 | 4 |
| 明白我的想法不必是正确的 | 0 | 1 | 2 | 3 | 4 |
| 更充分地享受生活中的小事 | 0 | 1 | 2 | 3 | 4 |
| 在心烦意乱时，平复我的情绪 | 0 | 1 | 2 | 3 | 4 |
| 停止对自己的负性（心理）冲动做出反应 | 0 | 1 | 2 | 3 | 4 |
| 发现困境中的积极方面 | 0 | 1 | 2 | 3 | 4 |
| 在承受压力时，减轻我的紧张感 | 0 | 1 | 2 | 3 | 4 |
| 意识到困境可以使我变得更强大 | 0 | 1 | 2 | 3 | 4 |
| 停止对当前状况毫无帮助的反应 | 0 | 1 | 2 | 3 | 4 |
| 觉察到美好的事情，并心存感激 | 0 | 1 | 2 | 3 | 4 |
| 放下不愉快的想法和感受 | 0 | 1 | 2 | 3 | 4 |
| 意识到自己的想法并非客观事实 | 0 | 1 | 2 | 3 | 4 |
| 留意到困境中那些让人开心的事 | 0 | 1 | 2 | 3 | 4 |
| 从不同的角度看待问题 | 0 | 1 | 2 | 3 | 4 |
